# ‘My child did not like using sun protection’: practices and perceptions of child sun protection among rural black African mothers

**DOI:** 10.1186/s12889-017-4688-7

**Published:** 2017-08-25

**Authors:** Zamantimande Kunene, Patricia N. Albers, Robyn M. Lucas, Cathy Banwell, Angela Mathee, Caradee Y. Wright

**Affiliations:** 1 Environment and Health Research Unit, South African Medical Research Council, Johannesburg, South Africa; 2 Environment and Health Research Unit, South African Medical Research Council, 1 Soutpansberg Road, Private Bag x385, Pretoria, 0001 South Africa; 3 National Centre for Epidemiology and Population Health, Research School of Population Health, Australian National University, Canberra, Australia; 4 Centre for Ophthalmology and Visual Sciences, University of Western Australia, Perth, Australia; 5 Department of Community Medicine, School of Public Health, University of the Witwatersrand, Johannesburg, South Africa; 6 Faculty of Health Sciences, University of Johannesburg, Johannesburg, South Africa; 7 Department of Geography, Geoinformatics and Meteorology, University of Pretoria, Pretoria, South Africa

**Keywords:** Solar ultraviolet radiation, Environmental health, Africa, Skin of colour

## Abstract

**Background:**

Photodamage is partially mitigated by darker skin pigmentation, but immune suppression, photoaging and cataracts occur among individuals with all skin types.

**Methods:**

To assess practices and acceptability to Black African mothers of sun protection equipment for their children living in a rural area, participants were recruited at the time of their child’s 18-month vaccinations. Mothers completed a baseline questionnaire on usual sun behaviours and sun protection practices. They were then provided with sun protection equipment and advice. A follow-up questionnaire was administered two weeks later.

**Results:**

Mothers reported that during the week prior to the baseline questionnaire, children spent on average less than 1 hour of time outdoors (most often spent in the shade). Most mothers (97%) liked the sun protection equipment. However, many (78 of 86) reported that their child did not like any of the sun protection equipment and two-thirds stated that the sun protection equipment was not easy to use.

**Conclusions:**

Among Black Africans in rural northern South Africa, we found a mismatch between parental preferences and child acceptance for using sun protection when outdoors. A better understanding of the health risks of incidental excess sun exposure and potential benefits of sun protection is required among Black Africans.

## Background

Solar ultraviolet radiation (UVR) induces deoxyribonucleic acid (DNA) damage and photoaging in all skin phototypes, including people with dark skin [1]. There is an inverse correlation between such damage and constitutive skin pigmentation [2]. Excess exposure to UVR is the main modifiable risk factor for skin cancer, causes some forms of cataract and can suppress immune responses [3]. While individuals with dark skin (or high levels of melanin in the skin) are relatively protected from the adverse effects on the skin of high exposure to solar UVR, they remain susceptible to both the eye and immune system effects [4, 5].

Children’s skin tend to be more sensitive to the sun due to a thinner stratum corneum [1, 6], and there is deeper penetration of UVR into the eye in children compared to adults [7]. Skin and eye damage can be prevented by adopting sun protection behaviours such as using sunscreen, umbrellas, wearing hats, wearing long-sleeve clothing, seeking shade and wearing sunglasses, as well as learning sun safe habits from an early age [6]. Parents and caregivers play an important role in the use of sun protection equipment by children [8]. Evidence shows that children who received encouragement from their parents and caregivers at an early age to adopt sun safe behaviour were more likely to continue using sun protection later in life [6]. Parents can encourage children by applying sun protection to their children or making sun protection equipment available to children who can protect themselves with minimal assistance [6].

Parents’ personal behaviours are strongly associated with their children’s sun protection [9]. Evidence shows that there is often a lack of understanding from parents on the importance of sun safe behaviour [10]. Few studies have looked at sun protection in children with dark skin. In general, these studies have shown that parents and/or caregivers tend not to encourage children to adopt sun safe behaviours including the use of sun protection equipment because of a perception that their child’s dark skin provides adequate natural protection from UVR [11, 12]. In Africa, no retrievable studies have considered mothers’ perceptions of the feasibility and acceptability of using sun protection equipment among small children with darkly pigmented skin. Instead, most African studies have focussed on the uptake of sun protection in children with oculocutaneous albinism (OCA), a genetically-inherited autosomal recessive condition in which individuals lack melanin and are therefore highly susceptible to the harmful effects of solar UVR [13, 14]. Here, we report the results of a survey of sun exposure behaviours and use of sun protection in mothers and children participating in a related study (described in detail elsewhere [15]).

## Methods

### Study setting and population

**Fig. 1:**
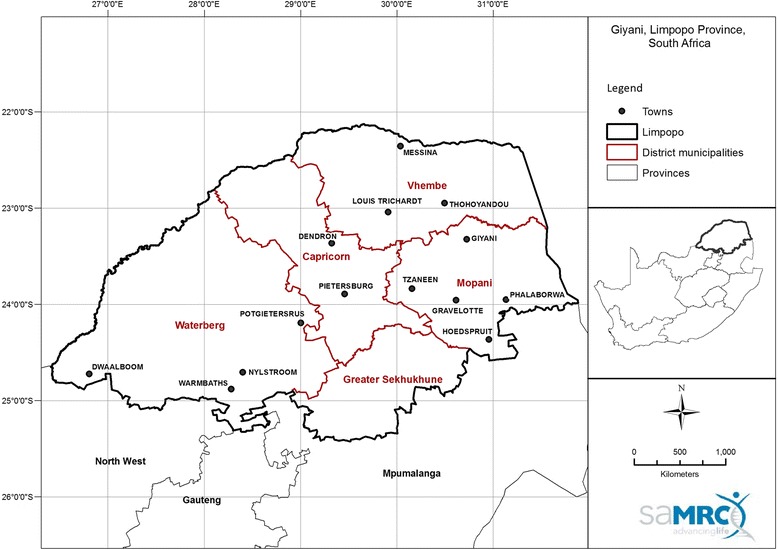
Location of the study clinics in Mopani District Municipality in the Limpopo Province of South Africa. The town of Giyani where the two study clinics were located is in the northern parts of the Mopani District Municipality. (The map was made in-house by the South African Medical Research Council)

The methods for the parent study from which these data derive (Clinical Trial Number: TRN PACTCR201611001881114, 24 November 2016, retrospective registration) have been described in detail elsewhere [15]. In brief, participants (*n* = 100) were recruited from two rural clinics (50 participants per clinic) in the Greater Giyani Local Municipality, Limpopo Province, South Africa (Fig. 1).

Officials from the Limpopo Provincial Department of Health provided the study team with the names and locations of all of the clinics in Greater Giyani. Several clinics were visited to establish their suitability as study sites, in terms of waiting room size, capability to provide space for research nurses. Suitable sites were similar in regard to size of the service community, demographic and economic factors of the community, training levels of the clinic staff, and having limited indoor waiting space. In addition, the study sites needed to be more than 25 km apart in order to minimise participants changing between the two clinics during the course of the study. The eligible sites were shortlisted and 2 sites were randomly selected for inclusion in the parent study.

### Study design and participant eligibility

The study took place during summer and early austral autumn from December 2015 to May 2016. Eligible participants were children receiving their 18-month vaccinations. Additional eligibility criteria included that the child had received the first measles vaccine, their mother, caregiver or guardian (hereafter called mother) was deemed able to comprehend the research and complete the sun exposure diary, and was capable of signing consent for the child to be enrolled in the study, the mother had a copy of the child’s Road to Health Chart, and the mother confirmed that they would be available for the duration of the study (4 weeks). Participant mother-child pairs were recruited consecutively; informed consent was obtained from mothers 18 years or older with children of 18 months or older. Mothers attending the clinics were provided with a sun protection intervention (see below for details of the sun protection intervention) and asked to use this with their child for 2 weeks.

Mothers of all child participants completed a baseline questionnaire. Mothers also completed a further questionnaire on the acceptability, use and uptake of the sun protection advice and equipment at 3–4 weeks after provision of the sun protection intervention, either face-to-face or by telephone. Interviews were conducted in Tsonga or Setswana and transcribed scripts were translated into English and then checked by a second Tsonga and Setswana-speaking researcher for correctness.

### Sun protection intervention

The intervention was provided to the mothers using a flyer and verbal explanation (according to a scripted protocol) where necessary. It included sun protection advice, namely to avoid the midday sun between 11 h00 and 14 h00, to seek shade whenever possible and to use the provided sun protection equipment. This included a hat, a long-sleeved top, an umbrella (to be held by the mother when carrying the child) and prior-researched effective sunscreen. The sun protection intervention, comprising equipment and advice, was provided to the mothers by the Research Nurses. In addition, each clinic received a gazebo to provide shade for attendees while they waited.

### Baseline questionnaire

The questionnaire was based on that used in an Australian study [16, 17] and tailored for local conditions. Key components of the questionnaire used in this analysis were a) general socio-demographic questions (i.e. role and age of adult participant; sex, population group and whether the child had OCA for the child participant); b) usual use of sun protection on the child; and c) child’s usual time spent outside. Specific sun-related questions are given in Table 1.

**Table 1:** Results of baseline questions on child’s usual time spent outside and usual use of sun protection for all children and by gender (Largest percentages in each comparison are noted in bold; Participants could select more than one response for questions 5 and 6)

		All children *n* = 98		Males *n* = 48		Females *n* = 50	
Section	Question and responses	n	%	n	%	n	%
Child’s time spent outside	1. Where does your child usually spend their time on weekdays:						
	Mostly inside	74	75.5	35	72.9	39	78.0
	Mostly outside	23	23.5	13	27.1	10	20.1
	*Missing*	1	1.0	0	0.0	1	2.0
	2. Where does your child spend most of their time on weekend days:						
	Mostly inside	66	67.4	26	54.2	40	80.0
	Mostly outside	28	28.6	20	41.7	8	16.0
	*Missing*	4	4.1	2	4.2	2	4.0
	3. If your child did spend time outdoors during daylight hours in the past week, was your child mostly in the shade or mostly out in the open in the sunshine:						
	Shade	91	92.9	43	89.6	48	96.0
	Open/sun	6	6.1	5	10.4	1	2.0
	*Missing*	1	1.0	0	0.0	1	2.0
	4. If your child did spend time outdoors during daylight hours in the past week, about how many hours does your child usually spend in the sunshine each day:						
	Less than 1 h	71	72.5	36	75.0	35	70.0
	About 1 h	14	14.3	5	10.4	9	18.0
	About 2 h	7	7.1	4	8.3	3	6.0
	About 3 h	1	1.0	0	0.0	1	2.0
	More than 3 h	3	3.1	3	6.3	0	0.0
	*Missing*	2	2.0	0	0.0	2	4.0
Use of sun protection	5. If your child did spend time outdoors during daylight hours in the past week, did your child usually use the following to protect their body from the sun:						
	Hat or cap	52	53.0	25	52.1	27	54.0
	Sunscreen	20	20.4	11	22.9	9	18.0
	Long-sleeved shirt	13	13.3	7	14.6	6	12.0
	Long pants or trousers	15	15.3	8	16.7	7	14.0
	Umbrella	49	50.0	22	45.8	27	54.0
	6. If you did apply sunscreen to your child’s body, which parts of the body was the sunscreen lotion applied:						
	Face	36	36.7	19	39.5	21	42.0
	Arms	5	5.10	3	6.2	6	12.0
	Legs	4	4.08	1	2.0	5	10.0
	Hands	1	1.02	1	2.0	3	6.0
	Back and shoulders	5	5.10	5	10.4	6	12.0
	Did not apply sunscreen to any body parts	51	52.0	25	52.0	28	56.0

### Follow-up questionnaire (post-intervention) administered face-to-face or by telephone

The follow-up questionnaire comprised the following questions as shown in Table 2: ‘what did you like about using the sun protection equipment on your child’; ‘was it easy to use the sun protection equipment’; ‘did your child like wearing/using the sun protection equipment we provided to you’; and ‘if your child didn’t like it, why not’. An open-ended question was included for ‘any further or additional comments or questions’.

**Table 2:** Responses to follow-up questions on the post-study use of sun-related equipment used by mother and child during the intervention study (Gender was missing for two children in the follow-up survey group)

	All children *n* = 88		Males *n* = 39		Female *n* = 47	
Question and responses	n	%	n	%	n	%
7. What did you think about using the sun protection equipment on your child:						
I liked it	86	97.7	38	97.4	46	97.8
I didn’t like it	2	2.2	1	2.5	1	2.1
I didn’t like the feeling of the sunscreen	4	4.5	1	2.5	3	6.3
I was worried the sunscreen would hurt my child	17	19.3	10	25.6	7	14.8
My friends or family thought I was crazy to put sun protection on my child	9	10.2	2	5.1	7	14.8
I did not like the umbrella	5	5.6	2	5.1	3	6.3
Other^a^	34	38.6	16	41.0	18	38.2
8. Was it easy to use the sun protection equipment:						
No	55	62.5	24	61.5	31	65.9
Yes	29	32.9	15	38.4	14	29.7
*Missing*	*4*	*4.5*	2	5.1	2	4.2
9. Did your child like wearing / using the sun protection equipment we provided to you?						
Yes	5	5.6	1	2.5	4	8.5
No	78	88.6	38	97.4	40	85.1
*Missing*	*5*	*5.68*	2	5.1	3	6.3
10. If your child didn’t like it, why not:						
My child didn’t want to wear any sun protection items	7	8.9	2	5.1	5	10.6
My child didn’t like the hat	18	23.0	8	20.5	10	21.2
My child didn’t like the sunscreen	6	7.6	3	6.9	3	6.3
My child didn’t like the long sleeve top	11	14.1	6	15.3	5	10.6

^a^Reasons given for ‘Other’ are provided in the section ‘*Responses to ‘Other’ for the question: “What did you think about using the sun protection equipment on your child”*

### Data analysis

Data from the questionnaires were manually coded and cross-checked by a second researcher, before being entered into Microsoft Excel [18] checked again and then transferred into STATA [19] for analysis. The responses to the open-ended questions were read by an experienced qualitative researcher (CB) who grouped them under two themes.. Simple frequencies and percentages were used for the quantitative data, including subgroup analysis according to the child’s sex, where possible.

## Results

Of the 100 mothers enrolled in the study, 98 completed the baseline questionnaire. Of those completing the baseline questionnaire, 10 mothers were not contactable to answer the follow-up questionnaire survey, providing an 89.8% response rate Of those completing both surveys, most of the adult participants were the child’s mother (*n* = 68, 77.3%), nine were the child’s guardian (10.2%) and seven were a family relation (7.95%) (four missing responses). The most common age group category for the adult participants was 18–25 years (44.3%), followed by 26–35 years (*n* = 21, 23.9%), 36–45 years (*n* = 17, 19.3%), 46–60 years (*n* = 10, 11.4%) and one participant was older than 60 years of age. Of the children enrolled in the study, 48 were boys, all were Black Africans and 14 were reported as having OCA.

### Child’s usual time spent outdoors and usual use of sun protection

Mothers reported that during the week prior to the baseline questionnaire, children spent on average less than 1 h of time outdoors; most time, on both weekday and weekend days, was spent indoors (Table 1, questions 1–6). When children did spend time outdoors during daylight hours in the previous week, they were most likely to spend time in the shade. Of the sun protection options offered, children were most likely to wear a hat or cap to protect their body from the sun. Most mothers did not apply sunscreen to their children and for those who did, they mostly applied the sunscreen to their child’s face. There were no statistically significant differences between boys and girls. When considering children reported to have OCA, five mothers stated that in the week prior to the baseline questionnaire they had applied sunscreen to their child’s face and seven stated that the child wore or a hat or cap when spending time outdoors.

### Follow-up questionnaire closed question responses

Survey responses regarding perceptions of sun protection equipment in Table 2 (questions 7–10) showed that of the 88 mothers who completed the follow-up survey, 86 liked the sun protection equipment. However, 78 mothers (89%) reported that their child did not like any of the sun protection equipment. For mothers who specified why the child did not like the equipment, 18 children did not like the hat (23%), 11 did not like the long-sleeve shirt (14%) and six did not like the sunscreen (8%). Nine mothers believed that their friends or family thought they were “crazy” to put sun protection on their child. Seventeen mothers (19.3%) reported that they were worried that the sunscreen would hurt their child (of the 86 who liked the sun protection equipment, 17 were worried that the sunscreen would hurt their child).

### Responses to ‘other’ for the question: “what did you think about using the sun protection equipment on your child?” and any other comments/questions

One theme concerned mothers’ responses to the immediate physical impact of sun protection equipment on their children. Many responded positively observing that “it had a good effect on my child”, “the child had no bad reaction”, “the child got no pimples” and “it stopped the child’s rash” (we did not assess what the rash was). One mother said, “(her) child did not change colour”, which probably indicated that the child did not get sunburnt. One mother said, “My child’s colour is looking good” while others said their children’s skin looked better after the sun protection intervention than before it. A few made negative comments such as her child got a rash from the sunscreen The other less commonly expressed theme was that mothers valued learning about the dangers of the sun for themselves and their children. It was expressed by this participant who said “I have learned about the sun and how to protect myself from the sun”. Mothers were also encouraged to comment and / or ask questions at the end of the follow-up survey (Table 3). Three mothers asked for how long they should continue to use the sunscreen.

**Table 3:** Open-ended responses and questions as they were mentioned by individual mothers and ranked by number of mentions

		Number of mothers *n* = 88	
	Response	n	%
Open-ended comments	I now know how to protect my children from sunburn	23	26.1
	One should protect children by using sunscreen	18	20.4
	I will not leave my child to play in the sun	9	10.2
	One should not get sunburned	8	9.0
	I was not aware that the sun can damage eyes	6	6.8
	I gained information about the harm the sun can do	6	6.8
	Children should not sit in the sun	5	5.6
	Children should wear sunscreen	3	3.4
	I now know protection is important	3	2.2
	Children should always be in the shade	2	2.2
	Children should drink water regularly	2	2.2
	I was not aware that children can get skin cancer	2	2.2
	I will continue using sunscreen	2	2.2
Questions	How long should I continue using the sunscreen?	3	3.4
	How does the sunscreen work?	1	1.1
	Where can we get sunscreen?	1	1.1
	Will my child be healthy if not exposed to the sun?	1	1.1
	I want to know more about how the sun can damage eyes?	1	1.1
	How does the equipment protect against the sun?	1	1.1
	Up to what age can the sun affect a child?	1	1.1

## Discussion

Black African mothers living in a sunny, rural part of South Africa reported that in the week prior to completing the baseline questionnaire, their 18-month old children were seldom outdoors and, if they were, they were mainly in the shade. Similarly, among 508 Caucasian Australian children aged 1 year of age 93% of mothers usually or always placed their child in the shade when they were outdoors [20].

The parent intervention study [15] from which our data were derived explored whether higher levels of sun exposure around the time of vaccination reduced the immune response to the vaccination (and therefore the level of protection against infectious disease). Our baseline data suggest that high levels of sun exposure in the week leading up to the vaccination were unlikely, with mothers and children largely staying indoors, possibly due to the relatively high temperatures that are experienced in Limpopo during summer months [21]. Nevertheless, high levels of incidental sun exposure may still have occurred, for example, from reflection from surfaces, through the tree canopy, and when walking to and from the clinic or market.

Previous reviews have found few reports for Black Africans exist on sun exposure [22] and photoprotection [22]. Those that do exist tend to focus on Black Africans with OCA. For example, hat-wearing among children with OCA (*n* = 90) attending a special needs school for those with visual impairment in northern South Africa was high (although brim width was not always sufficient) and only one third used sunscreen with an appropriate sun protection factor (SPF) rating [13]. Hat-wearing among 1-year old Caucasian children was also high (81%) as reported by their caregivers; although fewer caregivers put protective clothing (77%) and sunscreen (64%) on their children [20]. Hats were worn by about half of the children in our study; lower than that found in other studies. Among 2 to 4 year old Caucasian toddlers, caps were worn by 17% and hats by 63% of the study sample [16]. We provided hats to the mothers as part of the sun protection intervention and it is possible that this was the first occasion in the child’s life that they were told to wear a hat, although we did not confirm this.

Based on our follow-up questionnaire, although mothers generally liked the sun protection equipment, children did not like to use it. Mothers found it difficult to apply the sun protection, mainly because the children did not want to use it. This is in contrast to Australian mothers, where only 20% stated that they found it difficult to apply sunscreen [20]. Future studies could consider Black African mothers’ use of sun protection in addition to that of their child, since previous studies have found that mother’s use of sun protection may predict use of sunscreen for their children [20, 23–25]. No previous studies have evaluated these relationships among people with deeply pigmented skin. Most previous studies of how mothers sun protect their children have been among children with fair skin [26, 27]. In one large study of African-American adults [28] only one-third of participants engaged in at least one sun protective behaviour, with sunscreen being the least applied form of sun protection.

While mothers in the study sample liked the notion of sun protection and sun protection equipment, they may not have fully understood the reasons for its use. We gauged this from the open-ended responses provided by the mothers, such as ‘it stopped the child’s rash’. Other responses indicated that at least some of the mothers were keen to learn, i.e. ‘I now know how to protect my children from sunburn’. There is some evidence to suggest that sun protection awareness campaigns, such as the SunSmart Programme led by the Cancer Association of South Africa [29] do not reach all parts of South Africa and all population groups. These campaigns also focus on skin cancer and sunburn prevention; neither of which are health outcomes that affect people with dark skin in the same way as people with light skin.

Some mothers had concerns about the safety of sun protection equipment, despite the Research Nurses providing a full explanation at the recruitment phase, when the study was explained and sun protection measures were distributed. A small number of mothers reported that their friends or family thought they were crazy to put sun protection on their child. Sun safety campaigns targeting rural communities need to be holistic and comprehensive. They should include building knowledge about adverse sun-related health effects on the skin, eyes and immune system as well as embracing local issues such as where to buy affordable sunscreen and how to use it, and finding alternatives to conventional sun protection equipment such as natural, effective shade.

This study was part of a broader intervention study to assess whether higher levels of sun exposure diminish vaccination effectiveness in relation to immune responses against an infectious disease [15]. If that study does show an appreciable suppression of the immune response to the vaccination, there will be an imperative to better sun protect these rural-living African children around the time of vaccination. Our study suggests that this will require considerable work with communities to build confidence in the safety and use of sun protection, and its value, so that using sun protection is not stigmatized.

While the study data for the use of sun protection equipment by the child participants relied on mothers’ perceptions and report, and the sample size was relatively small (and we only focussed on one age group of children), we have provided some evidence of Black African mothers’ (in the current study site) perceptions of sun protection for their children and reasons for low uptake of sun protection equipment and practices in a rural community. Information bias may have influenced mothers’ like of the sun protection equipment and the cross-sectional nature of mothers’ report of children’s sun protection may require verification. Our study provides information on perceptions around use of sun protection in a rural environment for very young children. These results cannot be generalised to urban or semi-urban communities, or to children of other age groups. Similar studies in communities in different settings, and involving children of different age groups, would be of considerable interest.

## Conclusions

In summary, our results suggest that some Black African mothers in certain rural areas would like to try to make use of sun protection equipment on their children; however, barriers such as an unwilling child and not knowing whether sunscreen is safe, among others, pose challenges to the feasibility and acceptability of uptake for children. Given that skin cancer rates are far lower among Black Africans compared to people with fair skin [30] the reason to sun protect for Black Africans is likely to be related to prevention of cataract and immune suppression. As efforts continue to better understand the health impacts of solar UVR exposure on pigmented skin, so too should efforts to understand and raise awareness about the potential harm that excess sun exposure may have, even on people with dark skin, and the value of using sun protection should be explained.

## Abbreviations

DNA: deoxyribonucleic acid
OCA: oculocutaneous albinism
SPF: sun protection factor
UVR: *s*olar ultraviolet radiation
